# Impact of Myocardial Revascularization Method on Smoking Cessation: Coronary Artery Bypass Grafting *versus* Percutaneous Coronary Intervention

**DOI:** 10.21470/1678-9741-2017-0041

**Published:** 2017

**Authors:** Ricardo das Neves, Greicy Kelly Avila, Fernando de Barros Oliveira, João Augusto Ferraz de Sampaio

**Affiliations:** 1 Cardiovascular Surgery Department of Hospital Santa Lucinda, Sorocaba, São Paulo, Brazil.; 2 Hospital Unimed, Salto, São Paulo, Brazil.

**Keywords:** Myocardial Infarction, Myocardial Revascularization, Angioplasty, Balloon, Coronary, Angioplasty, Smoking, Tobacco Use Cessation

## Abstract

**Introduction:**

Smoking is a serious public health issue, being a precursor of heart disease and a predictor of sudden death due to myocardial ischemia. Major events in the patient's health can lead to radical changes in habits and the choice for different myocardial revascularization methods might differently impact smoking cessation and relapse.

**Objective:**

To study the rate and perpetuation of smoking cessation after myocardial revascularization comparing coronary artery bypass grafting (CABG) and percutaneous coronary intervention (PCI).

**Methods:**

Smokers submitted to myocardial revascularization were divided into CABG and PCI groups. The research was conducted through interviews at the Hospital Santa Lucinda outpatient clinic. Patients with smoking cessation longer than 90 days before hospital admission, combined procedures, hospital readmission before 360 days after discharge, cases of death at any time, and emergency procedures were excluded from the study. The start of the smoking cessation period was determined as just after hospital discharge, with a follow-up of 12 months.

**Results:**

The proportion of patients reporting smoking relapse was significantly lower in the CABG than in the PCI group at 30 (11.1% *vs.* 20.8%; *P*=0.039) and at 180 days (23.1% *vs.* 41.5%; *P*=0.002), but no differences were observed between the two groups at 360 days after hospital discharge (51.9% *vs.* 54.1%; *P*=0.719). High levels of nicotine dependence and passive smoking showed to be important predictors of smoking relapse in the long-term.

**Conclusion:**

The occurrence of a major surgical procedure seems to have beneficial psychological effects, representing an interesting setting for smoking cessation counseling to have higher chances of success.

**Table:** 

Abbreviations, acronyms & symbols	
CABG	= Coronary artery bypass grafting
CRP	= C-reactive protein
INCA	= Instituto Nacional de Cânce
PCI	= Percutaneous coronary intervention

## INTRODUCTION

Smoking is a serious public health issue, being a precursor of heart disease and a predictor of sudden death due to myocardial ischemia^[1]^. According to the Instituto Nacional de Câncer (INCA), passive smokers present a 24% higher risk of myocardial infarction compared to control patients^[2]^. Smoking is an of the most important public health problems characteristic only of the human species. The World Health Organization says that smoking should be considered a pandemic, as around five million people die each year as a result of tobacco-related diseases. Of the total deaths occurred, four million are in the male sex and one million in the female sex. In the year 2025, there will be 10 million deaths from tobacco use if there is no change in the current prevalence of smoking. Cigarette kills more than the sum of other preventable causes of death such as cocaine, heroin, alcohol, fires, suicides and AIDS in developed countries^[3]^.

Smoking cessation is one of the main interventions during the treatment of ischemic coronary disease. Smoking cessation, alone, results in a reduction of approximately 36% in the risk of death and 32% in the risk of non-fatal myocardial infarction^[4]^. The success in quitting cigarette addiction, however, is limited, with success rates varying from 10% to 25% after one year of smoking cessation^[5]^. According to the literature, coronary artery bypass grafting (CABG) is an important motivation factor for smoking cessation^[6]^.

Considering the hypothesis that there is a strong association between a major event in the patient's health and a radical change in habit, the aim of the present study was to investigate the rate and perpetuation of smoking cessation after two different myocardial revascularization methods, comparing the findings of CABG and percutaneous coronary intervention (PCI).

## METHODS

This cross-sectional study was based on data collected at Santa Lucinda Hospital, in the city of Sorocaba, Brazil. A total of 199 patients underwent CABG surgery and 768 patients underwent PCI, between January 2014 and August 2015. The inclusion criterion was smoking, independent of the time of addiction, being excluded patients with smoking cessation for more than 90 days prior to hospital admission, with combined approach with angioplasty followed by revascularization during hospitalization, death and rehospitalization in the selected period. After the selection of patients, the study has 108 patients who underwent CABG and 159 patients who underwent PCI, who were interviewed and submitted to a questionnaire about nicotine addiction through the Fagerström test, smoking cessation conditions with 30, 180 and 360 days after hospital discharge, cohabitation with other smokers, schooling, comorbidities, age, sex, and median income. When complementation data was helpful, patients were approached by telephone contact.

Patients were divided into two groups according to the type of revascularization procedure performed: PCI or CABG. Nicotine dependence was classified according to score on Fagerström test as very low to medium (1 to 5 points) or high to very high (6 or more points). Age was categorized as ≤60 or >60 years old. Monthly income was recorded as a function of the Brazilian minimum wage (R$937.00 (US$287.89) in January 1^st^ 2017). Patients living with smokers were considered to be exposed to passive smoking. The main outcome of the study was relapse of smoking and determined only by a single cigarette consumed again.

Data regarding all factors assessed was described for each group (Table 1). Number of people relapsing to smoking was summarized in absolute numbers and cumulative percentages (Table 2). Differences between the groups evaluated were assessed with chi-square tests for categorical data and with t-tests for continuous data. The occurrence of smoking relapse was presented with Kaplan-Meier survival curves, and differences in smoking relapse rates between PCI and CABG groups were evaluated with a log-rank test.

**Table 1:** Descriptive analysis of patients undergoing surgical revascularization (CABG) or percutaneous intervention (PCI).

		CABG	PCI	*P*-value
Age	Mean and standard deviation (years)	61.8±8.8	65.5±10.1	0.02
	Up to 60 years	44 (40.7%)	53 (33.3%)	0.217
	Over 60 years	64 (59.3%)	106 (66.7%)	
Sex	Male	83 (76.9%)	98 (61.6%)	0.009
	Female	25 (23.1%)	61 (38.4%)	
Systemic arterial hypertension		60 (55.6%)	69 (43.4%)	0.051
Diabetes mellitus		24 (22.2%)	47 (29.6%)	0.183
Fagerström test	Very low to medium	83 (76.9%)	115 (72.3%)	0.407
	High to very high	25 (23.1%)	44 (27.7%)	
Passive smoking		12 (11.1%)	28 (17.6%)	0.144
Education	Middle school	69 (63.9%)	114 (71.7%)	0.393
	High school	26 (24.1%)	31 (19.5%)	
	Higher education	13 (12.0%)	14 (8.8%)	
Average income	1-2 minimum wages	59 (54.6%)	97 (61.0%)	0.520
	2-3 minimum wages	36 (33.3%)	43 (27.0%)	
	>3 minimum wages	13 (12.0%)	19 (11.9%)	

CABG=coronary artery bypass grafting; PCI=percutaneous coronary intervention

**Table 2:** Relapse to smoking in patients undergoing surgical revascularization (CABG) or percutaneous intervention (PCI).

	CABG	PCI	*P*-value
30 days	12 (11.1%)	33 (20.8%)	0.039
180 days	25 (23.1%)	66 (41.5%)	0.002
360 days	56 (51.9%)	86 (54.1%)	0.719

CABG=coronary artery bypass grafting; PCI=percutaneous coronary intervention

Logistic regression models were applied for identifying factors associated with smoking relapse at each time point of the study: 30, 180 and 360 days. Initially the association between the assessed factors and smoking cessation was assessed in univariate models (Table 3). Subsequently, all factors associated with smoking cessation with a *P*-value <0.250 on the univariate models were included in multivariate models. Stepwise backward elimination was applied for selection of the best fitting model. The final model was restricted to variables with *P*-values <0.05 in the multivariate analyses (Table 4). Interaction between the evaluated factors was assessed by adding interaction terms to the models. If significant interactions were observed, stratified analyses were performed to better explore each observed interaction.

**Table 3:** Univariate logistic regression model.

		30 days			180 days			360 days		
		OR	95% CI	*P*	OR	95% CI	*P*	OR	95% CI	*P*
Procedure	CABG	1.00	-	-	1.00	-	-	1.00	-	-
	PCI	2.10	1.03-4.27	0.042	2.36	1.36-4.07	0.002	1.09	0.67-1.79	0.719
Age	Up to 60 years	1.00	-	-	1.00	-	-	1.00	-	-
	Over 60 years	5.66	2.15-14.89	<0.001	1.16	0.68-1.97	0.580	1.26	0.77-2.08	0.361
Sex	Male	1.00	-	-	1.00	-	-	1.00	-	-
	Female	1.35	0.69-2.62	0.382	1.23	0.72-2.10	0.458	1.09	0.65-1.83	0.740
SAH		1.42	0.75-2.70	0.288	0.72	0.43-1.19	0.200	0.91	0.56-1.47	0.693
DM		0.46	0.19-1.07	0.071	0.90	0.51-1.61	0.726	1.19	0.69-2.05	0.534
Fagerström test	Very low to medium	1.00	-	-	1.00	-	-	1.00	-	-
	High to very high	2.51	1.29-4.91	0.007	13.95	7.20-27.03	<0.001	6.32	3.19-12.50	<0.001
Passive smoking		0.36	0.11-1.21	0.099	1,95	0.99-3.85	0.055	4.26	1.88-9.63	0.001
Education	Middle school	1.00	-	-	1.00	-	-	1.00	-	-
	High school	0.79	0.36-1.77	0.570	1.03	0.55-1.92	0.927	1.34	0.73-2.45	0.342
	Higher education	0.16	0.02-1.24	0.08	0.80	0.33-1.94	0.623	0.73	0.32-1.63	0.438
Average income	1-2 minimum wages	1.00	-	-	1.00	-	-	1.00	-	-
	2-3 minimum wages	0.65	0.31-1.38	0.263	0.74	0.41-1.32	0.302	0.72	0.42-1.23	0.228
	>3 minimum wages	0.42	0.12-1.46	0.171	0.66	0.29-1.53	0.332	0.77	0.36-1.66	0.507

CABG=coronary artery bypass grafting; CI=confidence interval; DM=diabetes mellitus; OR=odds ratio; PCI=percutaneous coronary intervention; SAH=systemic arterial hypertension

**Table 4:** Multivariate logistic regression model.

		30 days			180 days			360 days		
		OR	95% CI	*P*	OR	95% CI	*P*	OR	95% CI	*P*
Procedure	CABG	1.00	-	-	1.00	-	-			
	PCI	2.15	1.00-4.62	0.05	2.84	1.46-5.51	0.002			
Age	Up to 60 years	1.00	-	-						
	Over 60 years	7.85	2.77-22.21	<0.001						
DM		0.39	0.15-1.00	0.05						
Fagerström test	Very low to medium	1.00	-	-	1.00	-	-	1.00	-	-
	High to very high	4.17	1.93-9.00	<0.001	15.23	7.62-30.43	<0.001	7.32	3.53-15.18	<0.001
Passive smoking								4.69	1.93-11.40	0.001

CABG=coronary artery bypass grafting; CI=confidence interval; DM=diabetes mellitus; OR=odds ratio; PCI=percutaneous coronary intervention

The present study was approved by the research ethics committee of Fundação São Paulo - PUC-SP Sorocaba campus and the participating patients provided written informed consent. Data were analyzed with IBM SPSS software version 24 (IBM, New York, USA) and GraphPad Prism 5 (GraphPad Software Inc., La Jolla, USA). All tests were two-sided and *P*-values lower than 0.05 were considered as statistically significant.

## RESULTS

In total, 159 patients who had PCI and 108 patients who had CABG were included. The characteristics of the patients and the procedures, as well as the variables analyzed in the study, are presented in Table 1. Patients from the PCI group were significantly older than those from the CABG group (65.5±10.1 *vs.* 61.8±8.8). The gender distribution was also significantly different between the two groups, with 76.9% of males in the CABG group and 61.6% of males in the PCI group. The prevalence of systemic diseases (systemic arterial hypertension and diabetes mellitus), the level of dependence to nicotine according to the Fagerström score, exposure to passive smoking, education level and monthly income were not significantly different between patients who had CABG or PCI.

**Fig. 1:**
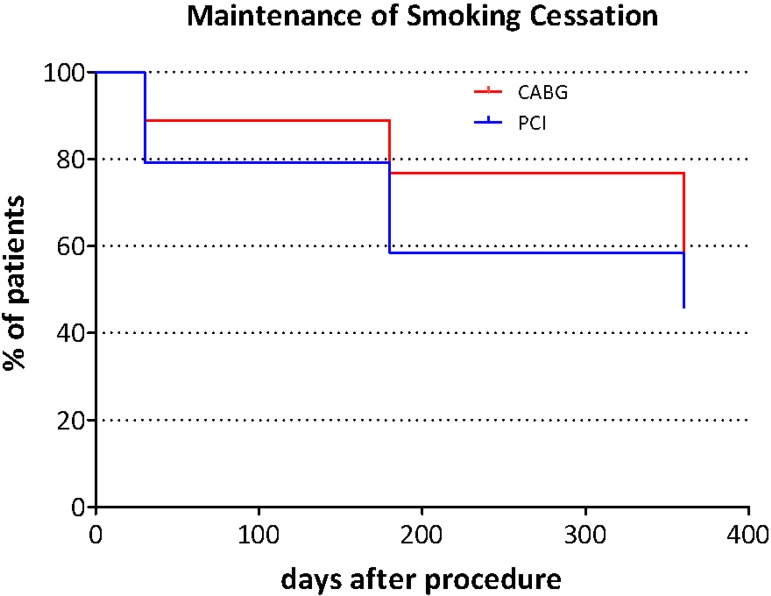
Kaplan-Meier curves for the maintenance of smoking cessation (log-rank test, P=0.238). CABG=coronary artery bypass grafting; PCI=percutaneous coronary intervention

The proportion of patients reporting smoking relapse was significantly lower in the CABG than in the PCI group at 30 (11.1% *vs.* 20.8%) and at 180 days (23.1% *vs.* 41.5%), but no differences were observed between the two groups at 360 days after hospital discharge (Table 2). The occurrence of smoking relapse in patients who had CABG and in those who had PCI was visualized with Kaplan-Meier survival curves (Figure 1). No significant differences on the occurrence of smoking relapse between the two groups was observed with the log-rank test (*P*-value=0.238).

Univariate logistic regression models indicated that the type of revascularization procedure was significantly associated with smoking relapse at 30 and at 180 days after hospital discharge, with patients from the PCI group being more likely to relapse smoking than patients from the CABG group (OR: 2.10, 95% CI: 1.03-4.27 and OR: 2.36, 95% CI: 1.36-4.07, respectively). At 360 days no association between type of revascularization and hospital discharge was observed.

Older age (>60 years old) was associated with a higher rate of smoking relapse 30 days after hospital discharge (OR: 5.66, 95% CI: 2.15-14.89), but not at later follow-up assessments. High levels of nicotine dependence assessed by the Fagerström score were associated with the occurrence of smoking relapse at all time points of the study (OR: 2.51, 95% CI: 1.29-4.91; OR: 13.95, 95% CI: 7.20-27.03 and OR: 6.32, 95% CI: 3.19-12.50 at 30, 180 and 360 days respectively). Passive smoking was not a significant predictor of smoking relapse at 30 and 180 days, but it was associated to smoking relapse at 360 days after hospital discharge (OR: 4.26, 95% CI: 1.88-9.63).

Multivariate logistic regression revealed that the association between type of revascularization procedure (CABG or PCI) and smoking relapse at 30 and 180 days was still observed even when taking possible confounders into account (OR: 2.15, 95% CI: 1,00-4,62 at 30 days and OR: 2.84, 95% CI: 1,46-5,51 at 180 days). The association between level of nicotine dependence and smoking relapse also remained significant, at all time points, after taking possible confounders into account (OR: 4.17, 95% CI: 1.93-9.00; OR: 15.23, 95% CI: 7.62-30.43 and OR: 7.32, 95% CI: 3.53-15.18 at 30, 180 and 360 days, respectively).

Significant interactions between the type of revascularization procedure and Fagerström score was observed in the multivariate models for 30 and 180 days of follow-up, while in the model for 360 days of follow-up a significant interaction between Fagerström score and passive smoking was observed. In order to better explore these interactions, additional analyses were performed, stratifying the cohort according to the variables between which interaction was observed.

At 30 days of follow-up, the type of intervention was significantly associated with smoking relapse among patients with high Fagerström scores (OR: 6.78, 95% CI: 1.52-30.25), but not among those with low Fagerström scores. On the other hand, Fagerström score was associated with smoking relapse among patients with PCI (OR: 7.16, 95% CI: 2.61-19.59), but not among those who had CABG. At 60 days of follow-up, similar results were observed when stratifying patients according to Fagerström score, with type of intervention being associated with smoking relapse only among patients with high Fagerström scores (OR: 9.23, 95% CI: 2.53-33.64). When stratifying patients according to type of intervention, however, a significant association between Fagerström score and smoking relapse was observed in both groups (OR: 6.41, 95% CI: 2,37-17,33 for CABG and OR: 34.23, 95% CI: 11.20-104.58 for PCI). At 360 days of follow-up, a correlation between Fagerström score and smoking relapse was only observed among patients not exposed to passive smoking (OR: 8.0, 95% CI: 3,67-17,45), while passive smoking was only associated with smoking relapse among patients with low/moderate dependence to nicotine according to Fagerström test (Tables 5 and 6).

**Table 5:** Stratified analysis of the interaction between variables "Procedure" and "Fagerström test" in the relapse of smoking.

**30 days**		**Fagerström test**		** **	** **	** **
		**Very low to medium**	**High to very high**	**OR**	**95% CI**	***P***
Procedure	CABG	9/83 (10.8%)	3/25 (12%)	1.36	0.31-6.00	0.683
	PCI	17/115 (14.8%)	16/44 (36.4%)	7.16	2.61-19.59	<0.001
	OR	1.16	6.78			
	95% CI	0.47-2.86	1.52-30.25			
	*P*	0.749	0.012			
**180 days**		**Fagerström test**		** **	** **	** **
		**Very low to medium**	**High to very high**	**OR**	**95% CI**	***P***
Procedure	CABG	12/83 (14.5%)	13/25 (52%)	6.41	2.37-17.33	<0.001
	PCI	23/115 (22.6%)	40/44 (90.9%)	34.23	11.20-104.58	<0.001
	OR	1.73	9.23			
	95% CI	0.82-3.67	2.53-33.64			
	*P*	0.154	0.001			

CABG=coronary artery bypass grafting; CI=confidence interval; OR=odds ratio; PCI=percutaneous coronary intervention

**Table 6:** Stratified analysis of the interaction between variables "Passive smoking" and "Fagerström test" in the relapse of smoking.

360 days		Fagerström test				
		Very low to medium	High to very high	OR	95% CI	*P*
Passive smoking	no	66/174 (37.9%)	44/53 (83%)	8.00	3.67-17.45	<0.001
	yes	19/24 (79.2%)	13/16 (81.3%)	1.14	0.23-5.62	0.872
	OR	6.2	0.89			
	95% CI	2.22-17.45	0.21-3.76			
	*P*	0.001	0.870			

CI=confidence interval; OR=odds ratio

## DISCUSSION

The present study addressed the hypothesis that there is a strong association between a major event in the patient's health and a radical change in habit, in this case smoking. We investigated the rate and perpetuation of smoking cessation after two different myocardial revascularization methods: CABG and PCI. Our results showed that the number of patients reporting smoking relapse was significantly lower in the CABG than in the PCI group up to 180 days of follow-up, but no differences were observed at 360 days after hospital discharge. Furthermore, although CABG represented a factor for smoking cessation in the short-term, high levels of nicotine dependence and passive smoking showed to be important predictors of smoking relapse in the long-term.

This study also showed an interaction between the myocardial revascularization method and the Fagerström score, in such a way that in patients with low levels of nicotine dependence, the procedure of choice has no significant impact on smoking cessation. This might be related to the fact that, in this group, most patients cease smoking regardless of the revascularization method, while in the group of patients with high levels of nicotine dependence mostly those undergoing CABG cease smoking and the ones subjected to PCI continue or relapse smoking.

Another interesting interaction found in the present study was between the Fagerström score and passive smoking. Our results suggest that in patients with low levels of nicotine dependence, passive smoking is related to a higher rate of relapse, while in patients with high levels of nicotine dependence passive smoking does not affect the maintenance of smoking cessation. For these patients, the rate of relapse is high regardless of the presence of a family member who also smokes, while for those, mostly the ones exposed to passive smoking will relapse smoking.

One limitation of the current study was that patients indicated to different revascularization procedures may also present different symptomatology, which could differently impact their predisposition to quit smoking and maintain smoking cessation. Future studies should take this variable into consideration. On the other hand, some of the strengths of the present study were its prospective nature and the long follow-up period. These two characteristics increase the reliability and validity of our findings.

Association between the procedure invasiveness and smoking cessation was also observed in another study with patients treated for coronary artery disease, in which a higher rate of smoking cessation was observed after surgical revascularization (55%) than after percutaneous intervention (25%) or angiography alone (14%) one year after the procedure^[7]^. A second study revealed comparable high rates of smoking cessation following surgical revascularization both at 60 days (93.15%) and 90 days (93.84%) after the procedure^[6]^. However, the proportion of patients who relapse 12 months after quitting smoking was similar in the percutaneous (53.4%) and surgical (52.7%) groups^[6]^. Several investigations through the past 15 years reported similar abstinence rates, with approximately 40% of smoking cessation maintenance at 12 months after myocardial infarction followed by elective surgical revascularization and 55.4% after percutaneous coronary intervention^[8,9]^.

Although patients submitted to major surgeries present a higher probability of succeeding on smoking cessation in the short-term, when compared to those undergoing a procedure in an outpatient setting, both this study and the literature show that this difference does not remain over time. An interesting alternative to potentiate the beneficial psychological effects of a major surgical procedure is the combination with counseling during hospitalization or at the time of surgical intervention, what was previously described as a teaching moment^[10]^. This way, an early intervention during hospital stay, could present a high possibility of success in smoking cessation^[11]^. Furthermore, if counseling is possible to be started a reasonable time before the intervention, it is also associated with lower rates of postoperative complications, as observed in a meta-analysis that revealed a 40% reduction in the relative risk of total complications with preoperative smoking cessation^[7,10,12-14]^.

Cigarette smoking represents a complex problem with significant implications for cardiovascular diseases and, although the prevalence of smoking decreased in the past 45 years, smoking cessation rates (defined as 6 months of tobacco abstinence) has been estimated at only 6% in recent years^[15]^.Cigarette smoking contributes to cardiovascular diseases in several ways. The toxic products of cigarette circulate in the bloodstream, increasing the inflammatory response and interfering with the function of the endothelium and causing abnormalities that lead to the development of atherosclerotic lesions on the arterial wall. This leads to the narrowing of blood vessels, gradually impairing blood flow, as well as makes the arteries stiffer, less elastic and prone to rupture^[16]^. Over time, it is possible to observe a progressively impaired vasomotor reactivity in smokers, as well as platelet dysfunction. Previous studies have described the correlation between smoking and increased levels of C-reactive protein (CRP), fibrinogen and homocysteine. Furthermore, smoking increases the inflammatory response, which in association with higher levels of homocysteine can represent an important mechanism of atherosclerosis development^[17]^.

Smoking cessation can reduce the progression of arterial disease, the risk of myocardial infarction, and the mortality due to cardiovascular diseases in more than one third, surpassing other preventive treatments^[9]^. When smokers are hospitalized they can be more prone to accept interventions for smoking cessation^[10]^, but individual characteristics, such as level of addiction to nicotine and contact with other smokers, are potential factors of failure^[18]^. Still, smoking cessation programs should be strongly encouraged for patients undergoing surgical procedures^[19]^.

## CONCLUSION

The present study showed that, in the short-term, the number of patients reporting smoking relapse was significantly lower among those undergoing CABG than in the group subjected to PCI in the short-term. However, in the long-term, this difference did not remain. High levels of nicotine dependence and passive smoking showed to be important predictors of smoking relapse. Still, the occurrence of a major surgical procedure seems to be a special setting for smoking cessation guidance to have higher chances of success. Further studies, combining counseling with the surgical intervention, should be performed to evaluate this scenario.

**Table:** 

Authors' roles & responsibilities	
RN	Conception of the work; acquisition, analysis and interpretation of data for the work; revising; final approval of the manuscript version to be published
GKA	Conception and design of the work; manuscript redaction or critical review of its content; final approval of the manuscript version to be published
FBO	Acquisition and analysis of data; final approval of the manuscript version to be published
JAFS	Acquisition and analysis of data; final approval of the manuscript version to be published

## References

[1] Cooke JP, Bitterman H (2004). Nicotine and angiogenesis: a new paradigm for tobacco-related
diseases. Ann Med.

[2] Instituto Nacional do Cancer Programa Nacional de Controle do Tabagismo.

[3] Araújo AJ, Menezes AMB, Dórea AJPS, Torres BS, Viegas CAA, Silva CAR (2004). Diretrizes para cessação do
tabagismo. J Bras Pneumol.

[4] Critchley JA, Capewell S (2003). Mortality risk reduction associated with smoking cessation in
patients with coronary heart disease: a systematic review.. JAMA.

[5] Barth J, Critchley J, Bengel J (2006). Efficacy of psychosocial interventions for smoking cessation in
patients with coronary heart disease: a systematic review and
meta-analysis. Ann Behav Med.

[6] Pietrobon RC, Barbisan JN (2010). Impact of coronary artery bypass graft surgery in smoking
cessation. Rev Bras Cir Cardiovasc.

[7] Rallidis LS, Lekakis J, Panagiotakos D, Fountoulaki K, Komporozos C, Apostolou T (2008). Long-term prognostic factors of young patients (≤35 years)
having acute myocardial infarction: the detrimental role of continuation of
smoking. Eur J Cardiovasc Prev Rehabil.

[8] Saksens NT, Noyez L (2010). Smoking behaviour and attitudes in patients undergoing cardiac
surgery. The Radboud experience. Interact Cardiovasc Thorac Surg.

[9] Hammal F, Ezekowitz JA, Norris CM, Wild TC, Finegan BA, APPROACH Investigators (2014). Smoking status and survival: impact on mortality of continuing to
smoke one year after the angiographic diagnosis of coronary artery disease,
a prospective cohort study. BMC Cardiovasc Disord..

[10] Pereira TV, Rudnicki M, Franco RF, Pereira AC, Krieger JE (2007). Effect of the G-308A polymorphism of the tumor necrosis factor
alpha gene on the risk of ischemic heart disease and ischemic stroke: a
meta-analysis. Am Heart J.

[11] Moghbeli N, Kirtane AJ, Ray KK, Murphy SA, Gibson CM, Braunwald E (2005). C-reactive protein and cardiovascular outcomes in smokers versus
nonsmokers in non-ST-elevation acute coronary syndrome (from the
TACTICS-TIMI 18 trial). Am J Cardiol.

[12] Guilbert JJ (2003). The world health report 2002 - reducing risks, promoting healthy
life. Educ Health.

[13] Azevedo e Silva G, Valente JG, Malta DC (2011). Trends in smoking among the adult population in Brazilian
capitals: a data analysis of telephone surveys from 2006 to
2009. Rev Bras Epidemiol.

[14] Sepehripour AH, Lo TT, McCormack DJ, Shipolini AR (2012). Is there benefit in smoking cessation prior to cardiac
surgery?. Interact Cardiovasc Thorac Surg.

[15] Centers for Disease Control and Prevention (CDC) (2011). Quitting smoking among adults: United States,
2001-2010. MMWR Morb Mortal Wkly Rep.

[16] Khullar D, Maa J (2012). The impact of smoking on surgical outcomes. J Am Coll Surg.

[17] Bazzano LA, He J, Muntner P, Vupputuri S, Whelton PK (2003). Relationship between cigarette smoking and novel risk factors for
cardiovascular disease in the United States. Ann Intern Med.

[18] Levitzky YS, Guo C-Y, Rong J, Larson MG, Walter RE, Keaney JF (2008). Relation of smoking status to a panel of inflammatory markers:
The Framingham offspring. Atherosclerosis.

[19] Yanbaeva DG, Dentener MA, Creutzberg EC, Wesseling G, Wouters EF (2007). Systemic effects of smoking. Chest.

